# Genome- Wide Analysis of the Nucleotide Binding Site Leucine-Rich Repeat Genes of Four Orchids Revealed Extremely Low Numbers of Disease Resistance Genes

**DOI:** 10.3389/fgene.2019.01286

**Published:** 2020-01-08

**Authors:** Jia-Yu Xue, Tao Zhao, Yang Liu, Yang Liu, Yong-Xia Zhang, Guo-Qiang Zhang, Hongfeng Chen, Guang-Can Zhou, Shou-Zhou Zhang, Zhu-Qing Shao

**Affiliations:** ^1^ Shenzhen Key Laboratory of Southern Subtropical Plant Diversity, Fairy Lake Botanical Garden, Shenzhen and Chinese Academy of Sciences, Shenzhen, China; ^2^ Institute of Botany, Jiangsu Province and Chinese Academy of Sciences, Nanjing, China; ^3^ VIB-UGent Center for Plant Systems Biology and Department of Plant Biotechnology and Bioinformatics, Ghent University, Ghent, Belgium; ^4^ State Key Laboratory of Pharmaceutical Biotechnology, School of Life Sciences, Nanjing University, Nanjing, China; ^5^ College of Life Sciences and Oceanography, Shenzhen University, Shenzhen, China; ^6^ Key Laboratory of National Forestry and Grassland Administration for Orchid Conservation and Utilization at College of Landscape Architecture, Fujian Agriculture and Forestry University, Fuzhou, China; ^7^ South China Botanical Garden, Chinese Academy of Sciences, Guangzhou, China; ^8^ College of Agricultural and Biological Engineering (College of Tree Peony), Heze University, Heze, China

**Keywords:** orchids, plant resistance genes, evolution, phylogeny, synteny

## Abstract

Orchids are one of the most diverse flowering plant families, yet possibly maintain the smallest number of the nucleotide-binding site-leucine-rich repeat (*NBS-LRR*) type plant resistance (*R*) genes among the angiosperms. In this study, a genome-wide search in four orchid taxa identified 186 *NBS-LRR* genes. Furthermore, 214 *NBS-LRR* genes were identified from seven orchid transcriptomes. A phylogenetic analysis recovered 30 ancestral lineages (29 *CNL* and one *RNL*), far fewer than other angiosperm families. From the genetics aspect, the relatively low number of ancestral *R* genes is unlikely to explain the low number of *R* genes in orchids alone, as historical gene loss and scarce gene duplication has continuously occurred, which also contributes to the low number of *R* genes. Due to recent sharp expansions, *Phalaenopsis equestris* and *Dendrobium catenatum* having 52 and 115 genes, respectively, and exhibited an “early shrinking to recent expanding” evolutionary pattern, while *Gastrodia elata* and *Apostasia shenzhenica* both exhibit a “consistently shrinking” evolutionary pattern and have retained only five and 14 *NBS-LRR* genes, respectively. *RNL* genes remain in extremely low numbers with only one or two copies per genome. Notably, all of the orchid *RNL* genes belong to the ADR1 lineage. A separate lineage, NRG1, was entirely absent and was likely lost in the common ancestor of all monocots. All of the *TNL* genes were absent as well, coincident with the *RNL* NRG1 lineage, which supports the previously proposed notion that a potential functional association between the *TNL* and *RNL* NRG1 genes.

## Introduction

Plants are exposed to the threat of pathogens on a day-to-day basis in their natural habitats. In order to survive, plants have developed systems to protect themselves from invading pathogens. Specifically, plants have evolved physical barriers like the surface composed of cuticle and wax, to block pathogens or the release chemical components like phenols, terpenes and compounds containing sulfur or nitrogen, to deter or dispose of invading enemies. Moreover, plants have an innate immune system for inducing rapid defense responses. This plant-specific immune system triggers a series of hypersensitive reactions after recognizing invading pathogens, resulting in apoptosis of infected cells, which halts the replication and spread of pathogen.

The core of this defending system involves a series of specific genes, namely, disease resistance (*R*) genes, which detect pathogens and trigger downstream resistance reactions. Five types of *R* genes have been discovered, including nucleotide-binding site and leucine-reach repeats (*NBS-LRR*), receptor-like protein (*RLP*), serine/theorine kinase (*STK*), receptor-like kinase (*RLK*) genes, and other genes that do not contain regular domains. Among all types of *R* genes, the *NBS-LRR* gene family is the largest and most important, containing over 60% of characterized *R* genes (Meyers et al., 2005; McHale et al., 2006; Friedman and Baker, 2007; Kourelis and van der Hoorn, 2018). This type of *R* genes originated early in the green plant lineage (Xue et al., 2012; Shao et al., 2019), and has expanded into a large gene family in angiosperms, usually consisting of hundreds of members in an individual genome. These members actively evolved with frequent recombinations occurring between paralogs, gene duplications and losses, and high substitution rates. Since the first genome-wide analysis was conducted on *NBS-LRR* genes in *Arabidopsis thaliana* (Meyers et al., 2003a), this gene family has been comprehensively studied across tens of plant genomes, most of which belong to the rosid lineage of the eudicots and Poaceae of the monocots (Yang et al., 2008; Porter et al., 2009; Li et al., 2010a; Lozano et al., 2012; Luo et al., 2012; Andolfo et al., 2013; Wu et al., 2014; Zhong et al., 2018).

*NBS-LRR* genes are divided into three classes, the *TIR-NBS-LRR* (*TNL*), *CC-NBS-LRR* (*CNL*) and *RPW8-NBS-LRR* (*RNL*), which are distinguished by the presence of a Toll/Interleukin-1 Receptor-like (TIR), coiled-coil (CC) or resistance to owdery mildew8 (RPW8) domain at the N-terminus of the translated proteins (Shao et al., 2016a; Shao et al., 2019). *RNL* genes were long considered to be part of the *CNL* genes due to some similarities between the sequences of the CC and RPW8 domains (Meyers et al., 2003b), but *RNL*s have too few members to be easily detected. Recently, a functional characterization study found that *RNL*s do not to function like regular *R* genes (Bonardi et al., 2011). A typical *R* gene, such as *TNL* or *CNL* genes, usually functions as a detector of certain pathogens and trigger resistance reactions, which is the beginning of the resistance pathway (McHale et al., 2006). The recognition of pathogens by the LRR domains of TNL and CNL proteins cause conformational changes in the NBS domain, which further promotes the multimerization of TIR or CC domains that transfer defense signals, while RNL proteins appear to be more downstream and transduce signals from the TNL and CNL proteins through an undetermined pathway (Bonardi et al., 2011). Nevertheless, *RNL*s are clearly indispensable in the resistance pathway, otherwise resistance would be affected (Peart et al., 2005). Evolutionary studies also found strong evidence that supports *RNL* genes as a new class of *NBS-LRR* genes, equivalent to *TNL* and *CNL* genes (Wu et al., 2014; Shao et al., 2016b; Qian et al., 2017). Interestingly, although both *CNL* and *RNL* genes are always present in monocots and dicots, *TNL* genes are absent from monocots, which is likely due to an ancient gene loss event upon the split of this lineage (Meyers et al., 2003a; Tarr and Alexander, 2009; Andolfo et al., 2013; Shao et al., 2016b; Zhang et al., 2017b).

Diverse evolutionary patterns of *NBS-LRR* genes have been observed in different angiosperm lineages. For example, both Fabaceae and Rosaceae exhibit a consistently expanding pattern (Shao et al., 2014; Jia et al., 2015); whereas Brassicaceae exhibits a pattern of expansion followed by contraction (Zhang et al., 2016); Solanaceae demonstrates complicated patterns, potato shows a “consistent expansion” pattern, tomato exhibits a pattern of “first expansion and then contraction,” and pepper presents a “shrinking” pattern (Qian et al., 2017). Despite the absence of *TNL*s, the number of *NBS-LRR* genes analyzed in monocot genomes comparable to that of eudicots. For example, Asian rice *Oryza sativa* possesses 498 *NBS-LRR* genes, outnumbering most eudicots (Li et al., 2010a; Shao et al., 2016b). The discrepancy of retained gene number is drastic among species. Maize (*Zea mays*), a species from the same grass family as rice, possesses no more than 140 *NSB-LRR* genes, which shows a four-fold discrepancy between the two species and suggests an active evolutionary mode of *NBS-LRR* genes in Poaceae. The evolutionary history of *NBS-LRR* genes in Poaceae has been comprehensively studied: Li et al. used *NBS-LRR* genes from four sequenced genomes (Asian rice, maize, *Sorghum bicolor* and *Brachypodium distachyon*) to reconstruct the evolutionary history of *NBS-LRR* genes, and compared the gene tree with the systematic relationship of these four species, reconciling 496 ancestral lineages in the grass family. Varying numbers of gene gain and loss events resulted in the gene number discrepancy across these four species, indicating a shrinking pattern in this family (Li et al., 2010b).

**Figure 1:**
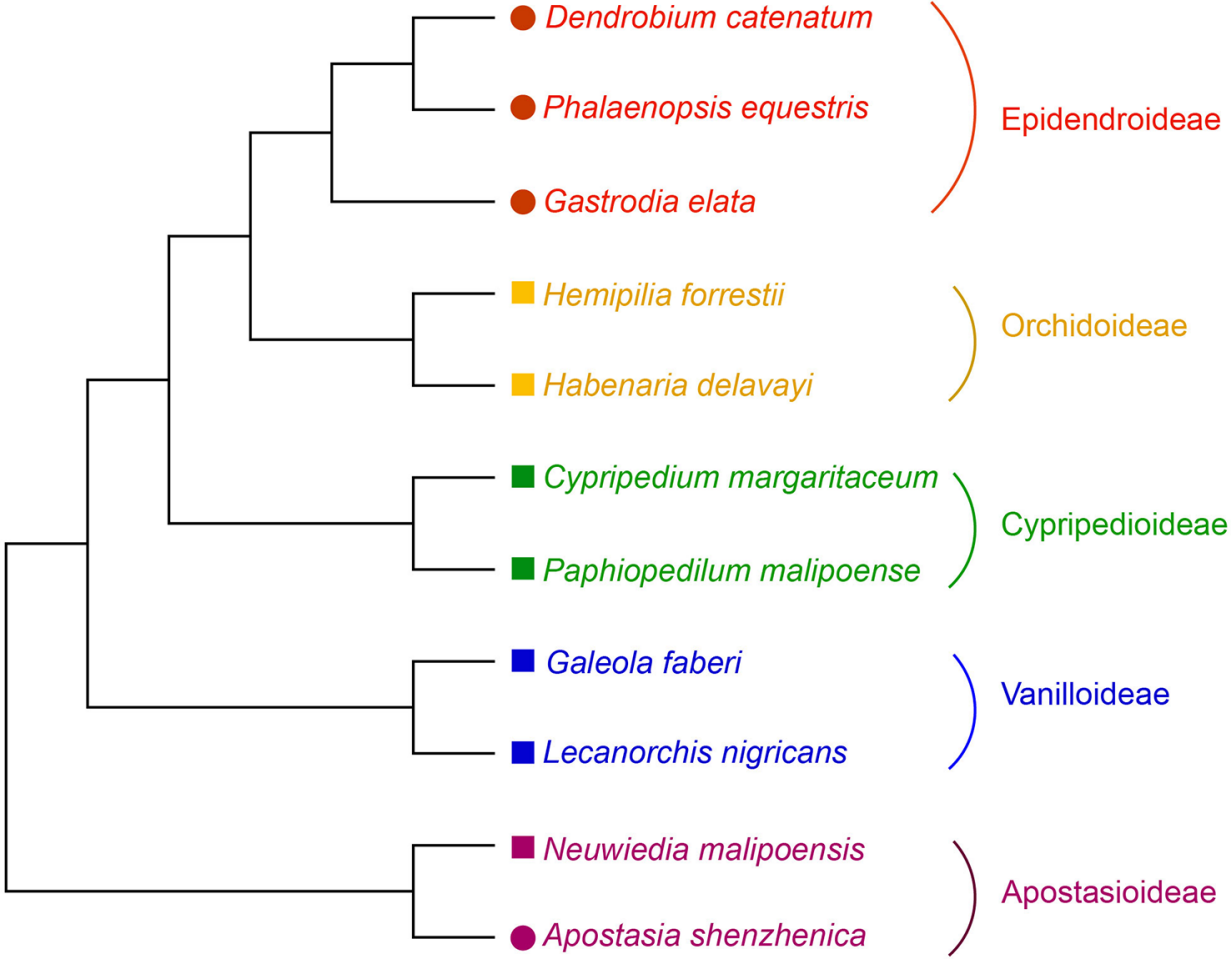
Phylogenetic relationship of orchid species used in this study. The phylogeny of the orchids used is from Zhang et al. (2017a). Species with genome or transcriptome information used in this study were indicated by colored solid circles or squares, respectively.

To date, only *NBS-LRR* genes of the grass family have been well studied in monocots. Whether or not other monocot lineages exhibit different evolutionary patterns remains unanswered, as the sequenced genomes are not as prevalent in other monocot lineages as in Poaceae. Fortunately, in recent years the sequenced genomes in Orchidaceae (orchids) have rapidly increased and multiple genomes of this family, another monocot lineage, have been made readily available. In this study, a genome-wide analysis of *NBS-LRR* genes in the four sequenced orchid genomes and seven orchid transcriptomes was conducted (**Figure 1**). The goal of this study was to uncover the evolutionary features and modes of *NBS-LRR* genes in this family and further investigate the mechanisms that have shaped these evolutionary changes.

## Results

### Identification and Domain Combination of *NBS-LRR* Genes From Four Orchid Genomes

A total of 186 *NBS-LRR* genes (**Table 1** and **Table S1**) were identified from four orchid genomes following previously described procedures (Xue et al., 2012; Shao et al., 2016b; Shao et al., 2019), among which, the *CNL* genes (182) overwhelmingly outnumbered *RNL* genes (4). *TNL* genes were absent from all four genomes, in accordance with the hypothesis that an early and thorough loss of *TNL*s had occurred upon the divergence of the monocot lineage (Tarr and Alexander, 2009; Shao et al., 2016b). *RNL* genes were found in three orchid genomes, except *G. elata*, but at extremely low numbers with one or two genes in each genome. Among the four orchids, the *D. catenatum* genome encoded the most *NBS-LRR* genes (115), followed by *P. equestris* (52) and *A. shenzhenica* (14), and *G. elata*, which encoded only five genes, the least among the four orchids and among all of the sequenced angiosperms. Since each orchid species had only one or two *RNL* genes, *CNL* genes must presumably be fully responsible for gene number variations among orchids.

**Table 1:** The number of identified *NBS-LRR* genes in four orchid genomes.

Domain compositions	*A. shenzhenica*	*G. elata*	*P. equestris*	*D. catenatum*
***CNL* class**	**13 (93%)**	**5 (100%)**	**50 (96%)**	**114 (99%)**
*CNL* (Intact)	2	2	**6**	23
*CN*	1	0	1	28
*NL*	8	0	11	13
*N*	2	3	32	51
***RNL* class**	**1 (7%)**	**0**	**2 (4%)**	**1 (1%)**
*RNL* (Intact)	1	0	2	1
*RN*	0	0	0	0
*NL*	0	0	0	0
*N*	0	0	0	0
**Total number**	**14**	**5**	**52**	**115**

Of the four orchids, intact *NBS-LRR* genes with all three domains (*CC/RPW8-NBS-LRR*) accounted for only 19.8% (37) of the total, whereas other genes either lacked a CC/RPW8 domain at the N-terminus, an LRR domain at the C-terminus, or lacked domains at both termini. *G. elata* had the highest proportion of intact genes (40.0%), while *P. equestris* had the lowest (15.4%). Several genomic changes, like recombination, fusion and pseudogenization, could result in real truncated genes, whereas other factors, such as sequencing, assembly errors and false annotations would elicit artificially “truncated” genes. Comparatively, the well-sequenced and annotated *A. thaliana* genome contains fewer (24.2%) truncated genes (Meyers et al., 2003b; Zhang et al., 2016).

### Conserved Motifs of the NBS Domain in Orchids

**Figure 2:**
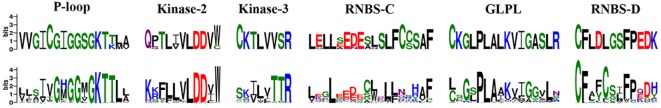
Conserved motifs in the NBS domain of the four orchid species. The amino acids of the six conserved motifs are extracted. Larger letters indicate higher frequency.

The NBS domain contains several smaller motifs of 10 to 30 amino acids in length. including P-loop, kinase 2, kinase 3, RNBS-C, GLPL, and RNBS-D (DeYoung and Innes, 2006). Using MEME and WebLogo, these motifs in orchid CNL and RNL proteins were identified (**Figure 2**). Although RNL proteins are conserved along the whole NBS domain, six motifs exhibited different extents of variation in CNL proteins. Differences between the CNL and RNL proteins were observed in all six motifs, especially kinase 3 and RNBS-C, which exhibited the greatest discrepancy. These motifs can be used to distinguish orchid *NBS-LRR* genes without conducting phylogenetic analyses.

### Phylogenetic Analysis of Orchid *NBS-LRR* Genes

**Figure 3:**
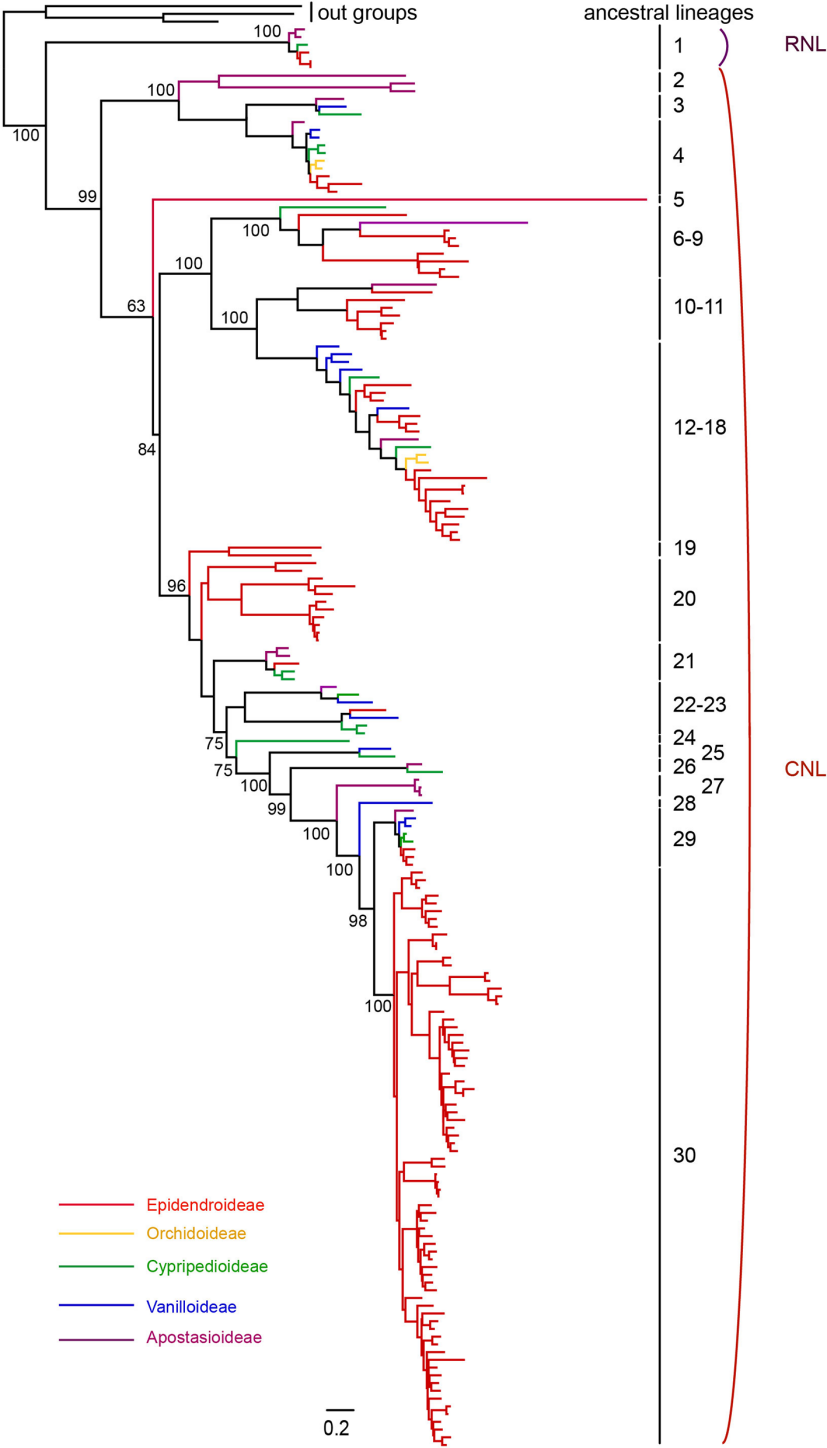
Phylogenetic relationships of *NBS-LRR* genes from orchid genomes and transcriptomes. *NBS-LRR* genes from different species are indicated with different colors in accordance to that of the species tree showed in **Figure 1**. Branch support values for two *NBS-LRR* classes (*CNL* and *RNL*) and each lineage are shown. The detailed phylogenetic tree is shown in **Figure S2**.

To explore the evolutionary history of *NBS-LRR* genes in orchids, a phylogenetic analysis using the protein sequences of the NBS domain was conducted using three *Amborella* TNL proteins as outgroups. In order to obtain a more complete evolutionary pattern of *NBS-LRR* genes in orchid, 214 *NBS-LRR* genes from seven orchid transcriptome were identified and involved for phylogenetic analysis (**Table S1**). The phylogeny revealed a deep divergence between the *RNL* and *CNL* genes, and the evolutionary rate of *RNL* genes was rather low, which was reflected by the short branches among species (**Figure 3**). Nevertheless, the branch separating *RNL* genes and *CNL* genes was long (**Figure 3**), supporting the hypothesis of ancient divergence between *RNL* and *CNL* genes.

Reconciling the phylogeny of orchid *NBS-LRR* genes by the species tree recovered 30 ancestral *NBS-LRR* lineages, including one *RNL* lineage and 29 *CNL* lineages. This represents the minimal number of ancestral *NBS-LRR* genes in the common ancestor of orchids, as the full *NBS-LRR* repertoire from the seven orchids could not be fully recovered from their transcriptomes. The reconciled *RNL* gene’s phylogeny was consistent with that of the orchid species tree, suggesting that they are descendants of one ancestral gene from the common ancestor and experienced no shared gene duplication (**Figures S1**and **S2**). *CNL* genes exhibited a more active evolutionary pattern with far more gene duplications and losses, as well as faster evolutionary rates, which was reflected by their longer branch lengths (**Figures S1** and **S2**). In total, 29 *CNL* lineages were identified from the orchid ancestor (**Figure 3**). Species-specific expansions were observed in different branches of the phylogenetic tree, with *D. catenatum*-specific expansion in Lineage 8, 9, 18, and 30, and *P. equestris*-specific expansion in Lineage 30 (**Figures 3** and **S2**). Moreover, independent gene losses occurred in the evolutionary history of the orchid family, thus, none of the four species maintained all ancestral lineages. Both gene duplications and losses have contributed to the gene number variations among the different species.

The phylogenetic tree shows that 30 ancestral *NBS-LRR* lineages were not all retained by all four orchids, but differentially kept by different taxa (**Table 2**). *D. catenatum* maintained 17 lineages, *A. shenzhenica* had 10, *P. equestris* had seven, and *G. elata* had three (**Table 2**). Interestingly, *P. equestris* retained fewer ancestral lineages than *A. shenzhenica*, but developed more genes. For the 30 recovered ancestral lineages, 21 of them were inherited by at least one analyzed genomes. Lineage 29 is the only one lineage retained by all four orchids, 11 lineages are inherited in only one taxon, five lineages are shared by two taxa, and four lineages are reserved in three taxa.

**Table 2:** Reservation of 30 ancestral *NBS-LRR* lineages in four orchids

Lineage	*D. catenatum*	*P. equestris*	*G. elata*	*A. shenzhenica*
1	**+**	**+**	**-**	**+**
2	**-**	**-**	**-**	**+**
3	**-**	**-**	**-**	**+**
4	**+**	**+**	**-**	**+**
5	**+**	**-**	**-**	**-**
6	**-**	**-**	**-**	**-**
7	**+**	**-**	**-**	**-**
8	**+**	**-**	**-**	**-**
9	**+**	**-**	**-**	**-**
10	**+**	**-**	**-**	**+**
11	**+**	**+**	**+**	**-**
12	**-**	**-**	**-**	**-**
13	**-**	**-**	**-**	**-**
14	**-**	**-**	**-**	**-**
15	**-**	**-**	**-**	**-**
16	**+**	**+**	**-**	**-**
17	**+**	**+**	**+**	**-**
18	**+**	**-**	**-**	**+**
19	**+**	**-**	**-**	**-**
20	**+**	**-**	**-**	**-**
21	**+**	**-**	**-**	**+**
22	**-**	**-**	**-**	**-**
23	**+**	**-**	**-**	**-**
24	**-**	**-**	**-**	**-**
25	**-**	**-**	**-**	**-**
26	**-**	**-**	**-**	**+**
27	**-**	**-**	**-**	**+**
28	**-**	**-**	**-**	**-**
29	**+**	**+**	**+**	**+**
30	**+**	**+**	**-**	**-**

“+” indicates the presence of the lineage, “-” indicates the absence of the lineage.

### Syntenic Analysis of *NBS-LRR* Genes in Orchid Genomes

**Figure 4:**
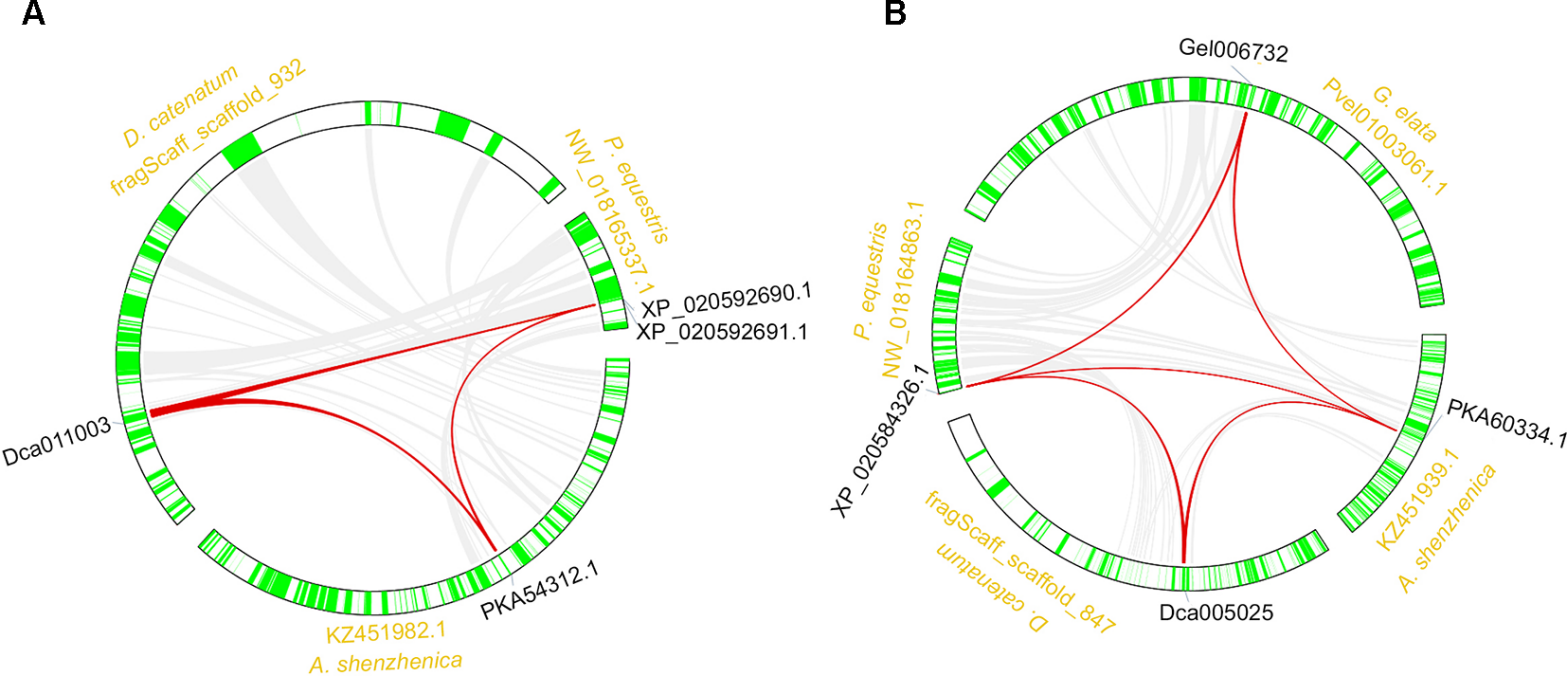
Synteny of orchid *NBS-LRR* genes. Syntenic genes of the *RNL* **(A)** and *CNL* **(B)** classes.

The synteny analysis was performed both between and within the four orchid genomes. Results revealed that the *RNL*s reserved synteny among three species, except *G. elata*, which lost the *RNL* genes (**Figure 4A**; **Table S2**). These results were in accordance with the synteny analysis of the *RNL* genes in other angiosperms and supported the conservative evolutionary pattern of this *NBS-LRR* subclass (Shao et al., 2016b). Synteny of the *CNL* genes was also detected for some conservatively evolved *CNL* lineages. Lineage 29 *CNL* genes from the four orchid genomes were detected on syntenic blocks (**Figure 4B**; **Table S2**).

Within genome synteny was used to determine which lineages of the *NBS-LRR* genes were derived from whole genome duplications (WGDs) or segmental chromosomal duplications (**Figure 4**). Surprisingly, no segmentally duplicated *NBS-LRR* genes were identified in the four genomes, whereas 47, 22, and 7 tandemly duplicated genes were detected in *D. catenatum*, *P. equestris*, and *A. shenzhenica*, respectively. The remaining *NBS-LRR* genes from the four genomes may have been duplicated from other duplication types. Although no segmentally duplicated *NBS-LRR* genes were identified based on the within genome analysis, the role of this duplication mechanism in *NBS-LRR* gene evolution could not be ruled out. First, the syntenic relationship of *NBS-LRR* genes would be disrupted during long-term evolution. Second, the segmentally duplicated *NBS-LRR* genes may have been lost during evolution. Therefore, the contribution of segmental duplication may be underestimated in the within genome synteny analysis.

### Reconciliation of Gene Losses and Gains and the Evolutionary Patterns in Orchids

**Figure 5:**
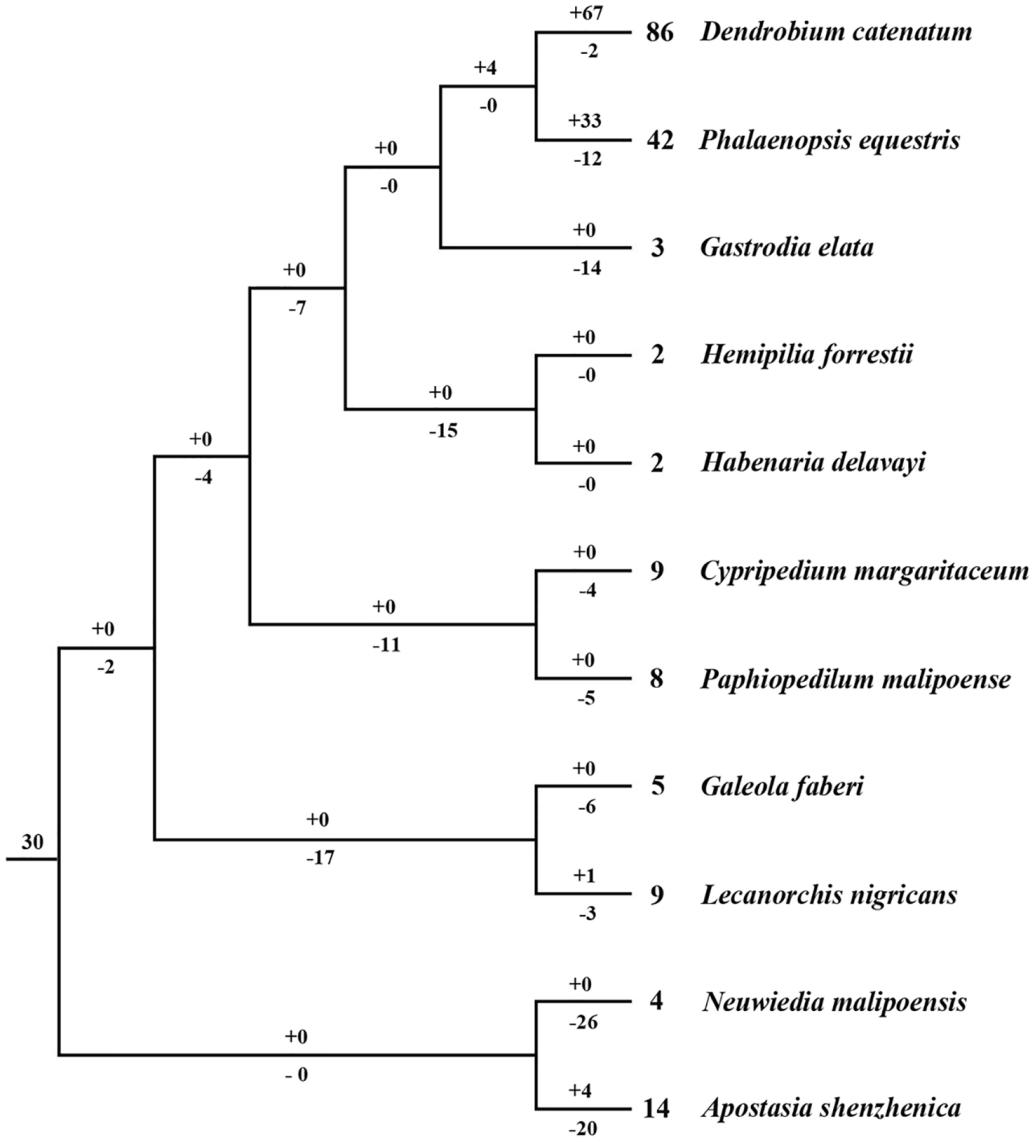
Loss and gain events of *NBS-LRR* genes across orchid evolution. Gene losses and gains are indicated by numbers with ‘–’or ‘+’ on each branch. Detailed information for gain and loss events of *NBS-LRR* genes is shown in **Figure S3**.

**Figure 6:**
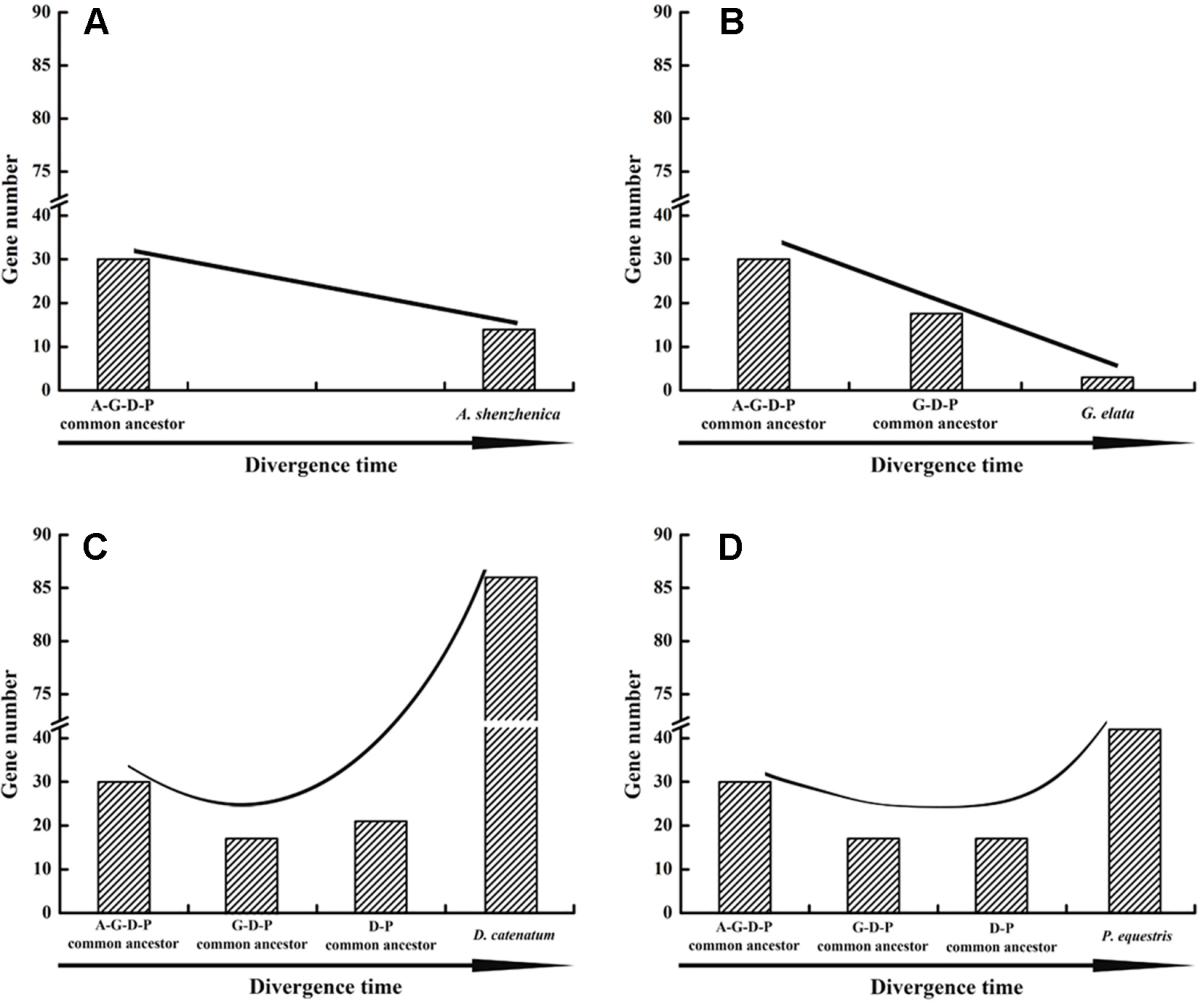
Evolutionary patterns of *NBS-LRR* genes in four orchid species. Evolutionary patterns of *NBS-LRR* genes in four orchids.: *A. shenzhenica* **(A)**, *G. elata* **(B)**, *P. equestris* **(C)**, and *D. catenatum* **(D)**. A-G-D-P indicates the common ancestor of all four orchids; GDP indicates the common ancestor of *G. elata*, *P. equestris* and *D. catenatum*, and D-P indicates the common ancestor of *P. equestris* and *D. catenatum.*

Based on the phylogenetic tree, it could be inferred that many independent gene gains and losses have occurred at different stages of orchid evolution (**Figure 5**). Starting from 30 ancestral genes, these four species have experienced considerably different evolutionary patterns: *A. shenzhenica*, the first split taxon, has undergone a process of more gene losses (20) than duplications (4), resulting in 14 *NBS-LRR* genes in its genome today. This basal taxon overall exhibits a shrinking pattern of evolution (**Figure 6**). The one taxon with fewer genes than the common ancestor, *G. elata*, should have experienced more severe gene losses. Before its divergence, the *NBS-LRR* genes in the common ancestor of *G. elata*, *D. catenatum* and *P. equestris* was reduced to 17 and *G. elata* experienced additional gene loss after its split. Thus, *G. elata* has undergone a “consistent shrinking” pattern (**Figure 6**). *D. catenatum* and *P. equestris* both have more genes than the common ancestor of orchids. Along their evolutionary trajectories, these two taxa have gained more genes than they have lost and recent independent duplications have made major contribution to the gene number increase in these two species. Based on the phylogenetic tree, it is clear that that species-specific duplications have expanded the gene numbers of lineage 8, 9, 18 and 30 in *D. catenatum*, and Lineage 30 in *P. equestris*, outnumbering the other two taxa. Therefore, *D. catenatum* and *P. equestris* both exhibit an “early shrinking to recent expanding” pattern (**Figure 6**). Overall, the four orchids exhibit two different patterns of *NBS-LRR* evolution, and the discrepancy depends on whether a given taxon underwent recent expansions.

**Figure 7:**
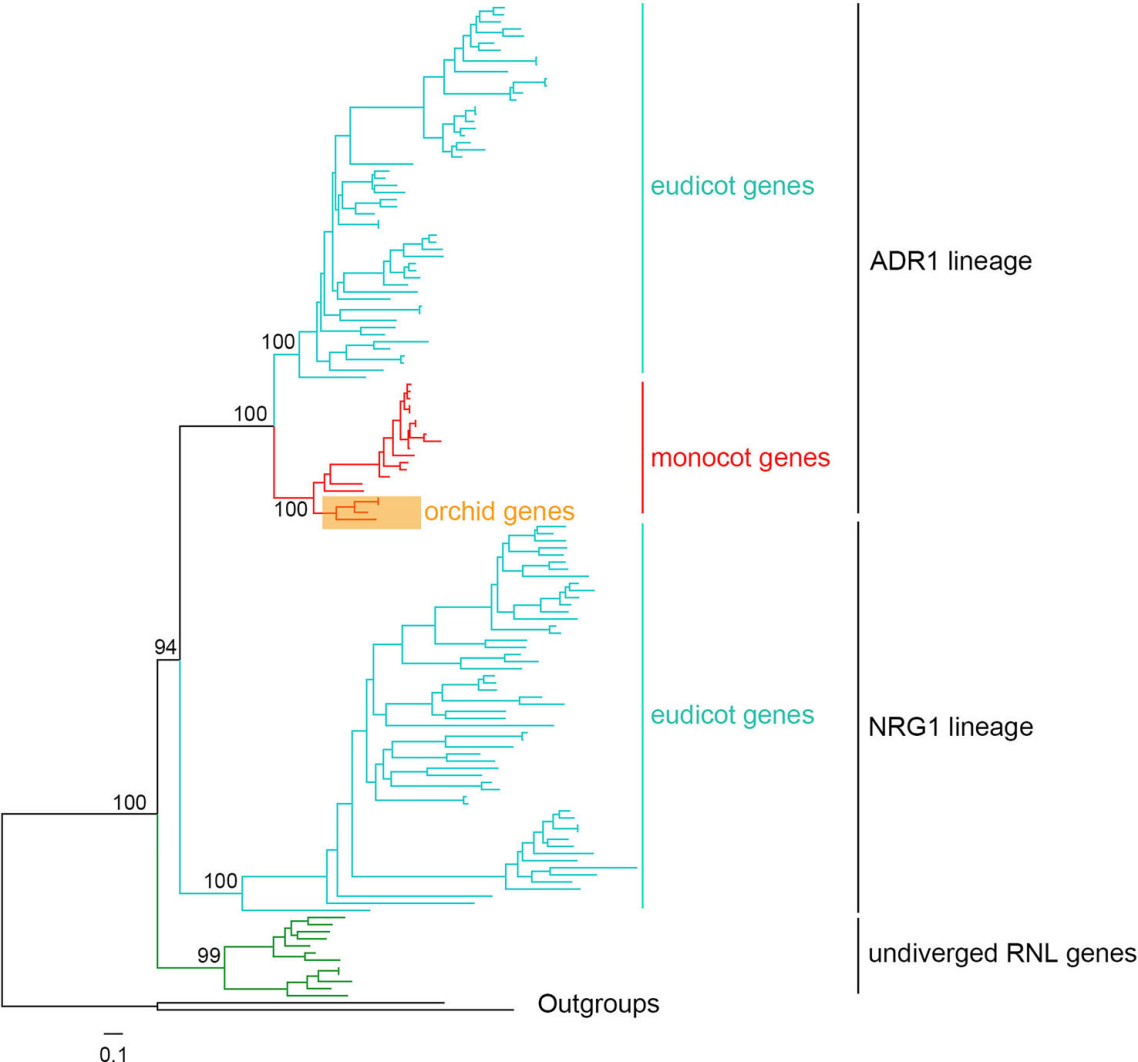
Phylogenetic tree of angiosperm *RNL* genes. A detailed phylogenetic tree of *RNL* genes is shown in presented **Figure S3**.

## Discussion

### The *NBS-LRR* Gene Number of Orchids

*NBS-LRR* genes belong to a large gene family in angiosperms, which includes hundreds of members. Only a small number of angiosperm taxa contain less than 100 *NBS-LRR* genes in their genomes. For example, Shao et al. analyzed 22 angiosperm genomes, which all had more than 100 *NBS-LRR* genes, except one Brassicaceae species, *Thellungiella salsuginea*, which had 88 genes (Shao et al., 2016b). In this study, it was discovered that three orchids also belong to the minority of plants that encode less than 100 *NBS-LRR* genes, and only one taxon, *P. equestris*, encoded over 100 genes. The number of *T. salsuginea NBS-LRR* genes fall below 100 because it underwent more severe gene loss events than duplications, after all, its ancestor once had d over 228 *NBS-LRR* genes (Zhang et al., 2016). The same situation was observed for three Cucurbitaceae species, *Cucumis sativus*, *C. melo*, and *Citrullus lanatus* (Lin et al., 2013). Orchids, however, are a different case. From a genetic and evolutionary perspective, as the reconciliation analysis suggests, the small number of orchid ancestral genes was mainly responsible for these results. In the orchid family, only 29 ancestral *CNL* genes and one *RNL* gene lineages were recovered as the family emerged, obviously far fewer than the 228 ancestral genes found in Brassicaceae (Zhang et al., 2016), 119 in Fabaceae (Shao et al., 2014), and 456 in Poaceae (Shao et al., 2016b). That’s why although *D. catenatum* and *P. equestris* have gained more genes than lost, they have not yet reached a large number of genes, such as rice or soybeans (Bai et al., 2002; Shao et al., 2014).

The number of *NBS-LRR* genes varies drastically among different taxa, even among closely-related species or subspecies. For instance, potato and tomato, both belonging to Solanaceae and have 447 and 255 *NBS-LRR* genes, respectively, showing a ratio of 1.75-fold difference in gene numbers (Qian et al., 2017). Intra-species variations of *Oryza*, *Glycine* and *Gossypium* reached a 5.4-fold discrepancy (Zhang et al., 2010). Therefore, the gene number variation observed in orchids. It is also noteworthy that the recent expansions are the main cause for this discrepancy was not surprising. Notably, recent expansions are the main cause for this discrepancy. In Fabaceae, Brasssicaceae and Solanaceae, the majority of expansions are consequence of tandem duplications (Shao et al., 2014; Zhang et al., 2016; Qian et al., 2017). In this study, *D. catenatum* and *P. equestris* appear to have undergone recent abrupt expansions, but mechanically tandem and ectopic duplications, other than WGDs are responsible for such expansions, as no syntenic genes were detected in these two species. *A. shenzhenica* and *G. elata* have not experienced sharp duplications, which explains the low number of *NBS-LRR* genes in these genomes. *A. shenzhenica* represents the earliest split of orchids, and has a rather narrow geographical distribution, as it is restricted to the Southeast Guangdong province, China (Zhang et al., 2017a). Its narrow distribution and stable habitat will likely lead fewer pathogen changes and stable pathogens diversity. Thus, *A. shenzhenica* has likely been battling a few of the same pathogens for a long period of time. Therefore, *A. shenzhenica* does not need to expand its *R* genes to face potential enemies. *G. elata*, despite its wide distribution, is an obligate mycoheterotrophic taxon, depending on a particular fungus *Armillaria mellea* to survive (Yuan et al., 2018), which probably does not allow *G. elata* to maintain many *R* genes. Coincidently, two other obligate mycoheterotrophic taxa, *Cuscuta australis* (Sun et al., 2018) and *Epipogium roseum* (unpublished), all seem to show a global gene loss pattern, and reduction of *R* gene number is only a part of the consequences.

### The Evolution of *RNL*s in Orchids and Other Angiosperms

According to a previous WGD, angiosperm *RNL* genes have diverged into two lineages, ADR1 and NRG1, based on *Aarbidopsis* and tobacco (Collier et al., 2011; Shao et al., 2016b). In this study, the comprehensive analysis of seed plant *RNL* genes revealed an undiverged clade of gymnosperm genes at the basal position, followed by two diverged clades, ADR1 and NRG1, in angiosperms (**Figures 7** and **S3**). Orchid *RNL* genes exclusively belong to the ADR1 lineage and have the shortest branch lengths among all of the angiosperms. Thus it is speculated that orchid *RNL* genes have been evolutionarily conserved since they have fewer diverse upstream signals to transduce. Orchids may be one of the plant lineages with the lowest number of *R* genes. The NRG1 lineage may have been lost as the origin of monocots, accompanied with the loss of an intron of the ADR1 lineage. The coincident loss of *TNL* genes and *RNL* NRG1 genes has been speculated to be due to their functional interdependence, as the resistance signals initiated by *TNL* genes are exclusively transduced by the NRG1 lineage (Collier et al., 2011). Several recent studies have suggested that nearly all test *TNL* genes are dependent on the *NRG1* gene for inducing hypersensitive reactions, although potential exceptions could exist (Qi et al., 2018; Castel et al., 2019; Wu et al., 2019). As a downstream gene with a conservative function, orchid *RNL* genes seem unnecessary to expand. Low copies are sufficient for maintaining a functional system. This may explain why *RNL* genes have remained in low numbers across the evolution of angiosperms.

## Materials and Methods

### Identification and Classification of *NBS-LRR* Genes

The whole genomes of four orchid taxa, *A. shenzhenica*, *C. elata*, *P. equestris* and *D. catenatum*, were used in this study. Genomic sequences and annotation files of *A. shenzhenica*, *P. equestris* and *D. catenatum* were downloaded from the NCBI database (https://www.ncbi.nlm.nih.gov/) (accession nos. PRJNA310678, PRJNA389183, and PRJNA262478, respectively). The genomic sequences and annotation files of *C. elata* were obtained from the *G. elata* Genome WareHouse Database (http://bigd.big.ac.cn/gwh/Assembly/129/show). The identification of *NBS-LRR* genes involved a two-step process. First, BLAST and hidden Markov model (HMM) searches using the NB-ARC domain (Pfam accession No.: PF00931) as a query, were performed simultaneously to identify candidate genes in each genome. For the BLAST search, the threshold expectation value was set to 1.0. For the HMM search (http://hmmer.org), default parameter settings were used. Second, all of the obtained candidate genes using BLAST or HMM searches were merged together, and the redundant hits were removed. The remaining candidate genes were submitted for an online Pfam analysis (http://pfam.sanger.ac.uk/) to further confirm the presence of the NBS domain with an E-value of 10^-4^. When two or more transcripts were annotated for a gene from alternative splicing, the longest form with an NBS domain was selected. All of the identified *NBS-LRR* genes were analyzed using the NCBI’s conserved domain database (https://www.ncbi.nlm.nih.gov/Structure/cdd/wrpsb.cgi) in order to determine the domains they possess.

### Sequence Alignment and Conserved Motif Identification

The amino acid sequences of the NBS domain were extracted from the identified NBS-encoding genes and used for multiple alignments using ClustalW (Tamura et al., 2011) and Muscle (Edgar, 2004) integrated in MEGA 7.0 (Kumar et al., 2016) with default parameter settings. NBS domain sequences that were too short (i.e., shorter than two-thirds of a regular NBS domain) or too divergent (i.e. genes whose NBS domains could not be well aligned with others, and the aligned lengths are shorter than two-thirds of a regular NBS domain) were removed to prevent interference with the alignment and subsequent phylogenetic analysis. Resulting amino acid sequence alignments were manually edited in MEGA 7.0 (Kumar et al., 2016) for further improvement. Conserved protein motifs were analyzed by the online programs MEME (Multiple Expectation Maximization for Motif Elicitation) and WebLogo (Crooks et al., 2004; Bailey et al., 2006) with default parameter settings.

### Phylogenetic Analysis and Reconciliation of Gene Loss/Duplication Events

In order to explore the relationships of *NBS-LRR* genes in the four orchids, a phylogenetic tree was reconstructed based on the aligned amino acid sequences of the conserved NBS domains. To avoid interference from “noisy characters,” too short or extremely divergent sequences were excluded from the phylogenetic analysis. Phylogenetic analyses were conducted using IQ-TREE and the maximum likelihood method (Nguyen et al., 2015). The best-fit model was estimated by ModelFinder (Kalyaanamoorthy et al., 2017). Branch support values were assessed with UFBoot2 tests (Minh et al., 2013). The scale bar indicated the genetic distance. *TNL* genes from the basal angiosperm, *Amborellla trichopoda*, were used as outgroups. Additionally, gene loss/duplication events during the speciation of the four orchid taxa were recovered by reconciling the *NBS-LRR* gene phylogenetic tree with the real species tree using Notung software (Stolzer et al., 2012). The phylogenetic analysis of *RNL* genes used the full length amino acid sequences of *RNL* proteins of 45 seed plants downloaded from Phytozome (https://phytozome.jgi.doe.gov/pz/portal.html).

### Syntenic Analyses Within and Across the Four Orchid Genomes

A synteny network approach was employed in this study (Zhao et al., 2017; Zhao and Schranz, 2019). Briefly, pair-wise all-against-all blast of protein sequences from the four genomes (*Apostasia*, *Gastrodia*, *Phalaenopsis* and *Dendrobium*) was performed. The obtained results and gff annotation files were then subjected to MCScanX for intra- and interspecies microsynteny detection and gene duplication type determination (Wang et al., 2012). Microsynteny relationship was displayed by TBtools (https://github.com/CJ-Chen/TBtools).

## Data Availability Statement

All datasets generated for this study are included in the article/**Supplementary Material**.

## Author Contributions

J-YX, Z-QS, S-ZZ and G-CZ conceived and designed the project. JYX, G-CZ, TZ and Z-QS obtained and analyzed the data. YL (3rd Author), YL (4th Author), Y-XZ, G-QZ, HC and S-ZZ participated in the data analysis and discussion. J-YX drafted the initial manuscript. Z-QS and YL (3rd Author) complemented the writing. All authors contributed to discussion of the results, reviewed the manuscript and approved the final article.

## Funding

This work was supported by grants from the Shenzhen Key Laboratory of Southern Subtropical Plant Diversity (SLPD-2018-3 to J-YX), the Jiangsu Key Laboratory for the Research and Uti1ization of Plant Resources (Institute of Botany, Jiangsu Province and Chinese Academy of Sciences, KSPKLB201835 to J-YX), and the Strategic Priority Research Program of Chinese Academy of Sciences (XDA13020603 to HC).

## Conflict of Interest

The authors declare that the research was conducted in the absence of any commercial or financial relationships that could be construed as a potential conflict of interest.

## Supplementary Material

Figure S1 A detailed ML phylogenetic tree with all sequence names and branch support values. The tree was reconstructed based on the NBS domain sequences of the *NBS-LRR* genes from the four genomes and seven transcriptomes.

Figure S2 Reconciled *NBS-LRR* gene tree with real species phylogeny and various loss and duplication events restored. “n3014” indicates a loss event that occurred in the common ancestor of Epidendroideae, Orchidoeae, Cypripedioideae, and Vanilloideae.

Figure S3 A detailed ML phylogenetic tree based on the full length sequences of *RNL* proteins from 45 seed plants.

Table S1 A list of NBS-LRR genes identified from seven orchid transcriptomes.

Table S2 Detailed information of the two synteny blocks.
