# Supplementary figures and images for: Genome- Wide Analysis of the Nucleotide Binding Site Leucine-Rich Repeat Genes of Four Orchids Revealed Extremely Low Numbers of Disease Resistance Genes
Source: Front Genet. 2020 Jan 8;10:1286. doi: 10.3389/fgene.2019.01286 (PMC6960632; doi:10.3389/fgene.2019.01286)

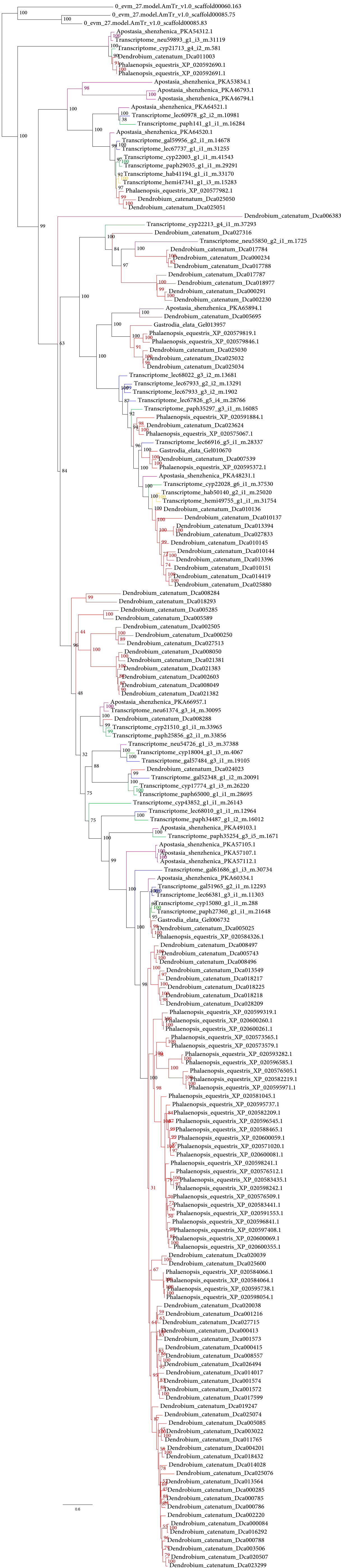

Supplement: Figure S1 — A detailed ML phylogenetic tree with all sequence names and branch support values. The tree was reconstructed based on the NBS domain sequences of the NBS-LRR genes from the four genomes and seven transcriptomes. [file Image_1.pdf]

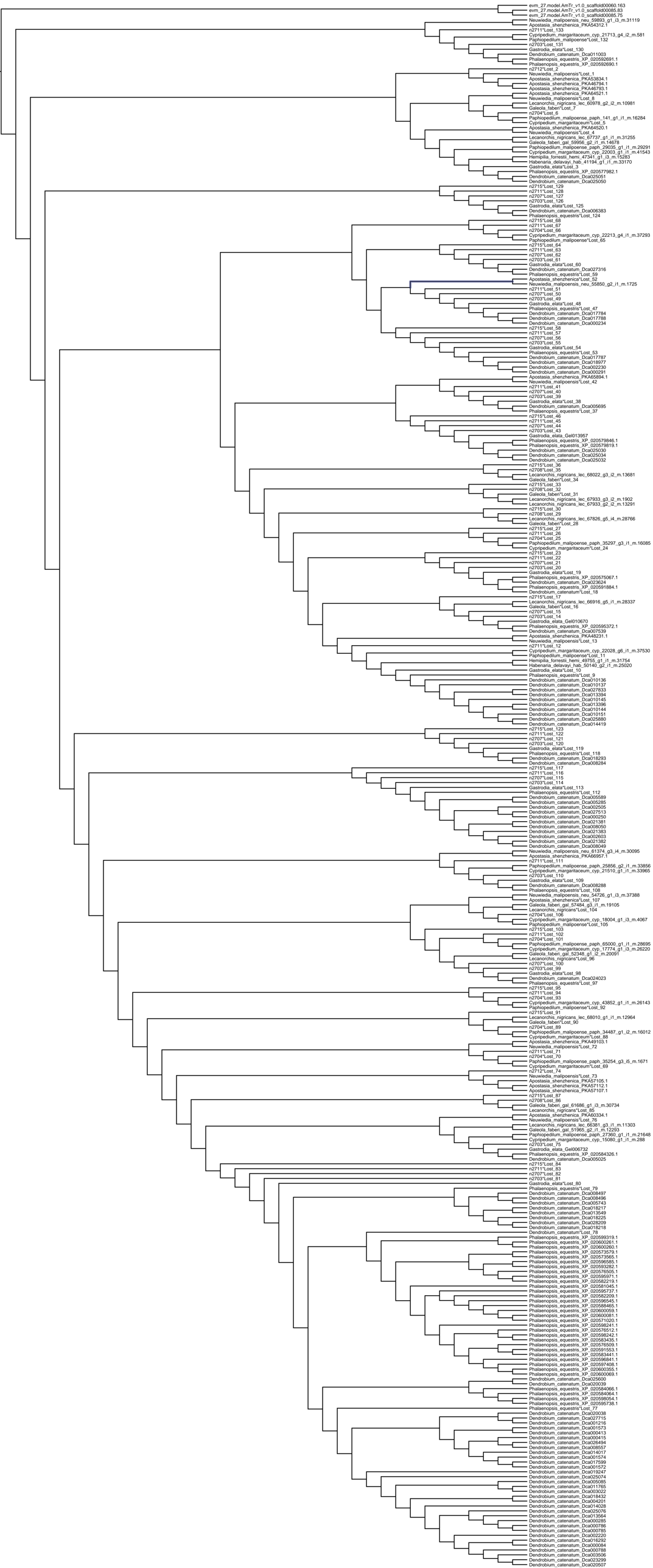

Supplement: Figure S2 — Reconciled NBS-LRR gene tree with real species phylogeny and various loss and duplication events restored. “n3014” indicates a loss event that occurred in the common ancestor of Epidendroideae, Orchidoeae, Cypripedioideae, and Vanilloideae. [file Image_2.pdf]

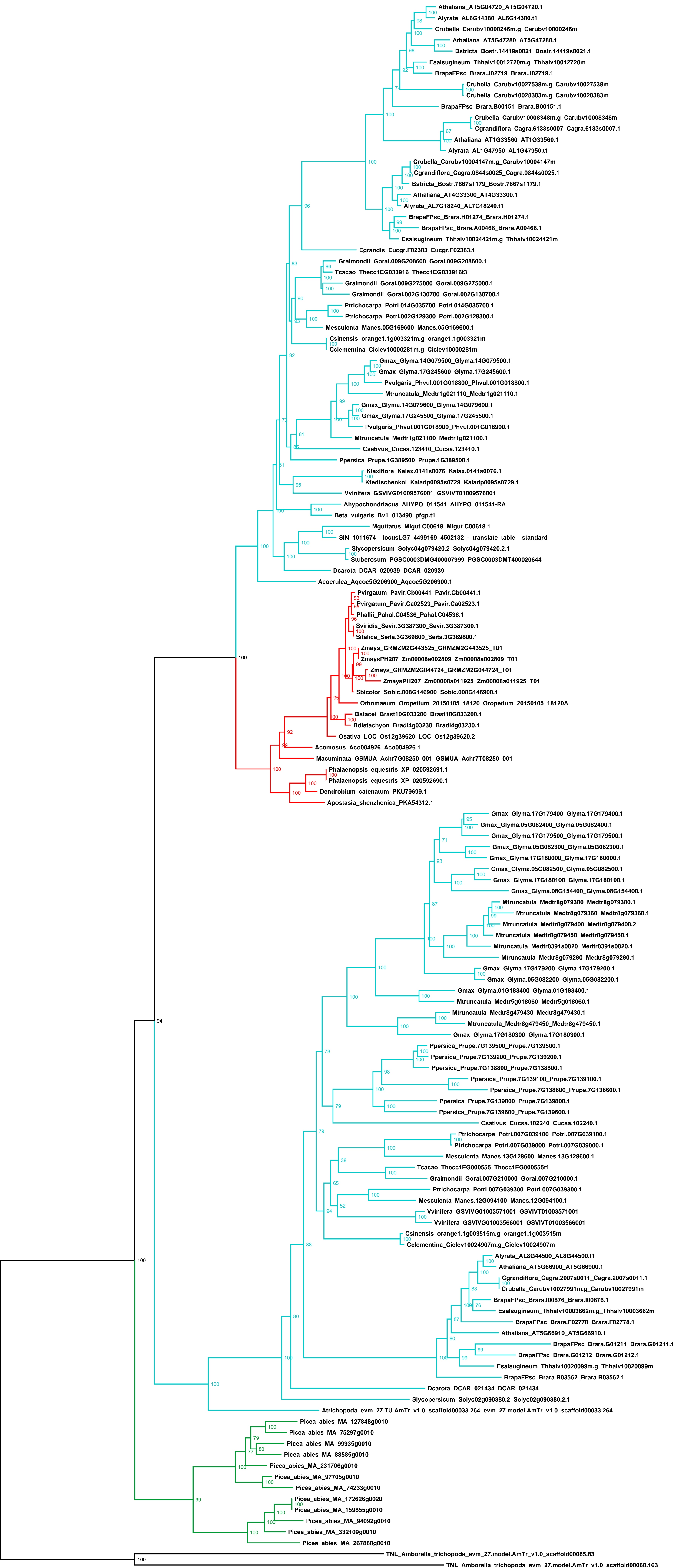

Supplement: Figure S3 — A detailed ML phylogenetic tree based on the full length sequences of RNL proteins from 45 seed plants. [file Image_3.pdf]
